# Longitudinal Neutrophil Cd64 Expression As a Biomarker For Acute Infection and Severity of Disease in Critically Ill Patients

**DOI:** 10.1186/2197-425X-3-S1-A521

**Published:** 2015-10-01

**Authors:** E de Jong, DW de Lange, A Beishuizen, ARJ Girbes, A Huisman

**Affiliations:** VU University Medical Center, Intensive Care, Amsterdam, the Netherlands; UMC Utrecht, Intensive Care, Utrecht, the Netherlands; UMC Utrecht, Department of Clinical Chemistry, Utrecht, the Netherlands

## Introduction

Cluster of Differentiation 64 (CD64) antigen is a membrane glycoprotein expressed on monocytes and macrophages. It is the high affinity neutrophil FcγRI receptor which is weakly expressed by neutrophils at physiological levels, but strongly upregulated within 4-6 hours by the pleiotropic cytokines such as Interferon-γ (IFN-γ) and Granulocyte Colony Stimulating Factor (G-CSF) which are produced in sepsis [1]. CD64 can be used as a diagnostic marker of bacterial infection and sepsis using flow cytometry.

## Objectives

The primary goal of this study was to determine whether CD64 is a useful biomarker for sepsis in adult critically ill patients. Secondly, since longitudinal data of CD64 expression are limited in the critically ill patient, a further goal was to clarify longitudinal expression patterns of CD64 on neutrophils with regard to outcome and sepsis severity.

## Methods

A prospective observational study between August 2011 and March 2013 in 155 consecutive patients who were admitted with sepsis, severe sepsis or septic shock was performed in a mixed medical-surgical ICU in a university hospital in the Netherlands. CD64 analysis were performed daily until discharge from ICU or death. Demographics, clinical, laboratory data and outcome defined as 28-day survival were recorded. They were included within 24 hours from start of antibiotic treatment.

## Results

Hundred-and-fifty-five consecutive patients were enrolled. Patients with a sepsis (1.53; 1.00-2.77) showed a lower CD64 index than patients with a severe sepsis and septic shock (2.66 ;1.36-4.49) p = 0.004. Furthermore a peak value on day two and subsequently a steady decline in all three groups was seen, independent of outcome.

A significant difference in CD64 at baseline of 2.65 [1.37-4.47] versus 1.74 [0.97-3.16] (p = 0.002) was seen between patients with a positive culture and negative culture at admission, respectively. This difference persisted until two days after inclusion. CD64 index was associated with severity in organ failure (SOFA-score). the higher the SOFA score, the higher the CD64 index measured at the same day. Analysis showed no significant difference during the first 14 days with regard to CD64 index for 28-day mortality between survivors versus non-survivors, although, towards the end, non-survivors are showing higher levels.

## Conclusions

This prospective study demonstrated that CD64 expression could be of diagnostic support, in addition to current tests in discriminating between critically ill patients with culture positive- and negative sepsis. Furthermore, CD64 expression correlates with severity of disease expressed as the daily SOFA-score, however, CD64 index was not a good predictor for 28-day mortality in the critically ill patient.

**Figure 1:**
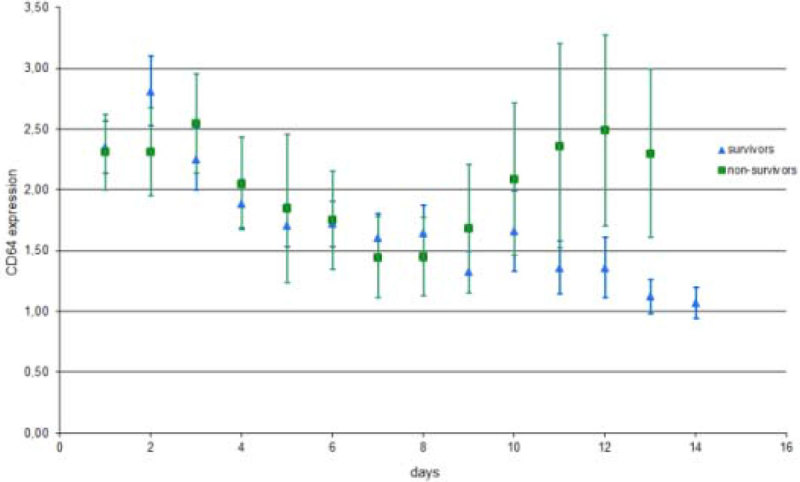
***Longitudinal CD64 expression in survivors versus non-survivors.***

**Figure 2:**
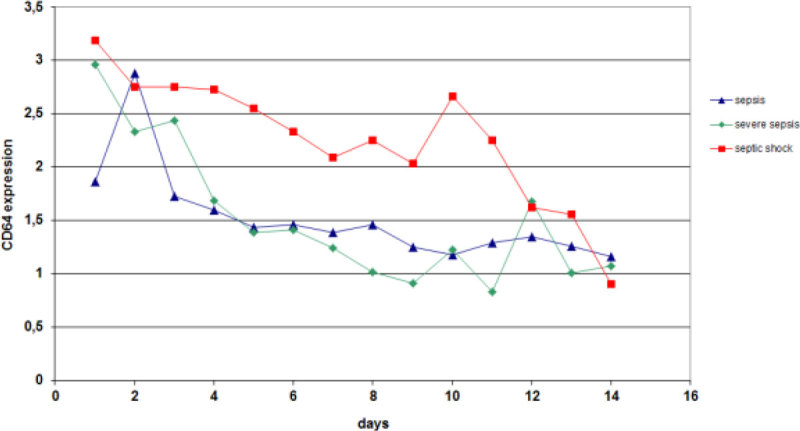
***Longitudinal CD64 expression in patients with sepsis, SS and shock.***
